# What Is the Significance of Unrecognized Non-Q-Wave Myocardial Infarction?

**DOI:** 10.1371/journal.pmed.1000060

**Published:** 2009-04-21

**Authors:** Clara Kayei Chow

**Affiliations:** 1 Population Health Research Institute, McMaster University/Hamilton Health Sciences, Hamilton, Ontario, Canada; 2 The George Institute for International Health, University of Sydney, Sydney, New South Wales, Australia

## Abstract

Clara Kayei Chow discusses the clinical implications of a new study that sought to examine the frequency and prognosis of unrecognized non-Q-wave myocardial infarction.

**Linked Research Article**

This Perspective discusses the following new study published in *PLoS Medicine*:

Kim HW, Klem I, Shah DJ, Wu E, Meyers SN, et al. (2009) Unrecognized non-Q-wave myocardial infarction: Prevalence and prognostic significance in patients with suspected coronary disease. PLoS Med 6(4): e1000057. doi:10.1371/journal.pmed.1000057

Using delayed-enhancement cardiovascular magnetic resonance, Han Kim and colleagues show that in patients with suspected coronary disease the prevalence of unrecognized myocardial infarction without Q-waves is more than 3-fold higher than that with Q-waves and predicts subsequent mortality.

## Background

Myocardial infarction (MI) that has no clinical symptoms (“silent MI”) or atypical symptoms is usually categorized as unrecognized MI (UMI). The mechanism underlying UMI is unclear, but given that silent MI is more common in people with diabetes, one hypothesis is that it may be related to autonomic neuropathy [1], [2]. UMI, which accounts for about one in four MIs in populations that are screened for events both clinically and with electrocardiogram (ECG), is a high-risk clinical condition associated with similar morbidity and mortality to clinically recognized MI [3], [4].

In most studies, UMI is identified through the incidental finding of Q-waves on ECG in patients who otherwise have no clinical symptoms or prior history. However, not all patients with MI develop Q-waves. In patients who initially present acutely with non-Q-wave MI, only about 15% will develop Q-waves [5]. Therefore it is likely that a substantial number of patients with UMI will be missed if the criteria for identifying MI is only Q-waves and nil else on ECG. Some studies therefore include other ECG abnormalities in their definitions, but while this may increase sensitivity for detection of UMI, it decreases specificity [6]. There are now more specific, albeit more expensive, tests for determining the presence of previous MI. Among these the identification of late gadolinium enhancement by cardiac magnetic resonance (CMR) appears to be sensitive and specific for myocardial scarring, and positive findings are predictive of poor prognosis [7].

## Non-Q-Wave UMI

In this issue of *PLoS Medicine*, Han Kim and colleagues report findings from a study that sought to examine the frequency and prognosis of non-Q-wave UMI [8]. The researchers prospectively recruited 185 patients with suspected coronary disease, but no clinical history of MI, who had been referred for invasive angiography. Patients who had a medical record of prior clinical MI, previous coronary revascularization procedure, or history of non-ischemic myocardial disorders that could cause myocardial scarring were excluded. Patients with serious intercurrent illness (e.g., cancer) that could shorten their lifespan to less than two years or who had a contraindication to CMR were also excluded.

All patients had CMR examination to determine the presence of delayed enhancement abnormalities consistent with previous MI and infarct size, and also underwent ECG and invasive coronary angiography. The prevalence of non-Q-wave UMI, defined as having no Q-waves on ECG but delayed enhancement abnormalities on CMR, was 27% (50/185), compared with a prevalence of 8% (15/185) for Q-wave UMI. In other words, in this cohort of patients about one third (65/185) had evidence of UMI by delayed enhancement on CMR, among whom 77% (50/65) had no Q-waves. For both Q-wave and non-Q-wave UMI patient groups, infarct size was small, and left ventricular ejection fraction (LVEF) was preserved for the majority of patients in both groups.

## Prognosis of Non-Q-Wave UMI

In Kim and colleagues' study, 16 deaths occurred in about two years of follow up: 13 in patients with non-Q-wave UMI (26%), one in a patient with Q-wave UMI (7%), and two in patients without MI (2%). Given that the numbers of patients with Q-wave UMI was low, it is probably incorrect to draw any conclusions from the apparent difference in mortality between the non-Q-wave and Q-wave UMI groups. However, patients with non-Q-wave UMI appeared to have more established cardiovascular risk factors and more severe angiographic findings of coronary artery disease (in terms of the extent and the proportion with obstructive disease).

Using multivariable analysis (including other significant predictors of mortality, New York Heart Association class, and LVEF in the model), non-Q-wave UMI was an independent predictor of all-cause mortality (hazard ratio 11.4, 95% confidence interval: 2.5–51.1) and cardiac mortality (hazard ratio 17.4, 95% confidence interval: 2.2–137.4). A measure of coronary artery disease severity was not included in these models, although baseline risk factors and revascularization were. The hazard ratios were significant, but had wide confidence intervals due to the small size of the study—thus the hazard ratios in those with non-Q-wave UMI in this study probably should not be generalized. The general finding of an association with adverse outcomes in this group is consistent with one other study of 195 patients screened with CMR that found that unrecognized myocardial scarring was associated with increased risk of adverse cardiac events [9].

## Clinical Implications

This important new study has two key clinical implications. First, previous non-Q-wave UMI is potentially being missed in patients with suspected coronary artery disease. Second, non-Q-wave UMI is important because it is significantly associated with increased mortality.

Patients with previous non-Q-wave UMI are difficult to identify, as they may not all be identified with usual clinical examination and tests. ECG findings may be normal or non-specific, and echo testing may not identify small or sub-endocardial infarcts with preserved LVEF. Myocardial viability studies would be a more sensitive and specific means of identifying patients that fall into this group, but these examinations are not routinely done, particularly in patients that already have invasive coronary angiography planned. The new study raises the question: should myocardial viability studies be done in all patients with suspected coronary artery disease? If these patients are receiving invasive coronary angiography and revascularization based on the findings, would knowledge of myocardial viability change their clinical management? It seems unclear at this time that viability testing should be routinely evaluated in all patients that have suspected coronary artery disease. Further research needs to be done to examine the overall effectiveness and costs associated with a strategy of routine viability testing.

How generalizable are Kim and colleagues' findings? The results are from a small select group of patients and therefore the prevalence rates quoted are unlikely to be representative of the general population, nor the population of patients that may be referred for invasive coronary angiography in the United States. The findings do indicate that non-Q-wave UMI is a frequent finding in high-risk patients with coronary artery disease.

Further studies need to be done to evaluate the determinants of the increased mortality in patients with non-Q-wave UMI and identify whether any additional treatments may result in prevention of future adverse outcomes.

## Abbreviations

CMR: cardiac magnetic resonance
ECG: electrocardiogram
LVEF: left ventricular ejection fraction
MI: myocardial infarction
UMI: unrecognized MI

## References

[1] Airaksinen KE (2001). Silent coronary artery disease in diabetes—A feature of autonomic neuropathy or accelerated atherosclerosis?. Diabetologia.

[2] Marchant B, Umachandran V, Stevenson R, Kopelman PG, Timmis AD (1993). Silent myocardial ischemia: Role of subclinical neuropathy in patients with and without diabetes.. J Am Coll Cardiol.

[3] Kannel WB, Abbott RD (1984). Incidence and prognosis of unrecognized myocardial infarction. An update on the Framingham study.. N Engl J Med.

[4] Sigurdsson E, Thorgeirsson G, Sigvaldason H, Sigfusson N (1995). Unrecognized myocardial infarction: epidemiology, clinical characteristics, and the prognostic role of angina pectoris. The Reykjavik Study.. Ann Intern Med.

[5] Kleiger RE, Boden WE, Schechtman KB, Gibson RS, Schwartz DJ (1990). Frequency and significance of late evolution of Q waves in patients with initial non-Q-wave acute myocardial infarction. Diltiazem Reinfarction Study Group.. Am J Cardiol.

[6] Ammar KA, Kors JA, Yawn BP, Rodeheffer RJ (2004). Defining unrecognized myocardial infarction: A call for standardized electrocardiographic diagnostic criteria.. Am Heart J.

[7] Kwong RY, Sattar H, Wu H, Vorobiof G, Gandla V (2008). Incidence and prognostic implication of unrecognized myocardial scar characterized by cardiac magnetic resonance in diabetic patients without clinical evidence of myocardial infarction.. Circulation.

[8] Kim HW, Klem I, Shah DJ, Wu E, Meyers SN (2009). Unrecognized non-Q-wave myocardial infarction: Prevalence and prognostic significance in patients with suspected coronary disease.. PLoS Med.

[9] Kwong RY, Korlakunta H (2008). Diagnostic and prognostic value of cardiac magnetic resonance imaging in assessing myocardial viability.. Top Magn Reson Imaging.

